# Acoustic therapy for allergic rhinitis and chronic rhinosinusitis: modulating microbiome, immunity and well-being

**DOI:** 10.3389/falgy.2025.1649031

**Published:** 2025-08-25

**Authors:** Jude Oluwapelumi Alao, Kelvin E. M. Lau, David White, Kevin Lee, Chris Puli'uvea, Jim Bartley

**Affiliations:** ^1^ School of Public Health and Interdisciplinary Studies, Auckland University of Technology, Auckland, New Zealand; ^2^ Centre of Chiropractic Research, New Zealand College of Chiropractic, Auckland, New Zealand; ^3^ School of Science, Auckland University of Technology, Auckland, New Zealand; ^4^ Department of Surgery, University of Auckland, Auckland, New Zealand

**Keywords:** nasal disease, vibration, allergic rhinitis, chronic rhinosinusitis, nitric oxide, nasal microbiome

## Abstract

Allergic rhinitis (AR) and chronic rhinosinusitis (CRS) are common respiratory conditions that significantly impact patient health and contribute to substantial healthcare burdens. While conventional treatments offer symptom relief, many patients continue to experience persistent symptoms, side effects, or resistance to standard therapies. This highlights the growing need for novel, non-invasive, and sustainable therapeutic strategies to manage chronic airway inflammation. This review examines acoustic therapy, an emerging non-pharmacological treatment that uses sound wave-induced vibrations as a potential adjunctive therapy for AR and CRS. Acoustic therapy shows potential benefits, including enhanced nitric oxide production, improved mucociliary clearance, and modulation of immune responses by activating mechanosensitive pathways and disrupting pathogenic biofilms. Preliminary clinical findings across some trials have reported improvements in peak nasal inspiratory flow ranging from approximately 17% to 31%, significant reductions in nasal congestion and symptom scores, such as Total Nasal Symptom Score, Sino-Nasal Outcome Test-22 (SNOT-22), and enhancements in sleep quality and patient-reported outcomes. Given this limited but expanding body of evidence, we integrate interdisciplinary insights from respiratory medicine, immunology, and microbiome science to provide a translational framework for future research. We highlight the need for rigorously designed clinical trials to assess acoustic therapy's therapeutic efficacy, safety, and long-term impact. As this field evolves, acoustic therapy holds significant potential to address unmet needs in chronic respiratory diseases and contributes to improved patient care.

## 1 Introduction

Chronic inflammatory nasal diseases, such as allergic rhinitis (AR) and chronic rhinosinusitis (CRS), represent a significant health challenge globally, impacting millions of people and creating substantial strain on healthcare systems. AR is characterized by an overreaction to inhaled allergens, leading to symptoms like sneezing, nasal congestion, itching, and a runny nose (1, 2). It often occurs alongside conditions such as asthma and atopic dermatitis. CRS, on the other hand, is characterized by persistent inflammation of the nasal and sinus mucosa, with different clinical presentations, especially in patients with chronic rhinosinusitis with polyps (CRSwNP) and without nasal polyps (CRSsNP). These conditions have similar underlying immune and inflammatory processes, including imbalanced cytokine signaling, microbial disruptions, and weakened mucosal immunity (3).

Traditional treatments, including antihistamines, intranasal corticosteroids (INCS), immunotherapy, and surgery, can provide symptom relief. However, they are often associated with side effects, varying levels of effectiveness, and difficulties in maintaining long-term improvements (4–8). The limitations of these conventional treatments highlight the need for novel therapies that target the root causes of these conditions more effectively. Recent advancements have introduced non-pharmacological treatments aimed at modulating the immune response and restoring microbial balance (9–11), reflecting a shift toward more patient-focused approaches in respiratory care.

One such promising treatment is acoustic therapy. Using sound wave-induced vibrations, acoustic therapy has been found to enhance the production of nitric oxide (NO), which plays a crucial role in supporting mucociliary clearance, promoting vasodilation, and bolstering antimicrobial defenses (12–14); mechanisms often disrupted in AR and CRS. Additionally, acoustic therapy can potentially reduce bacterial biofilms and restore the microbial balance within the nasal cavity (15, 16), which may address the treatment resistance commonly seen in CRS.

Despite promising results, acoustic therapy remains under-researched, particularly regarding its effects on the nasal microbiome, its impact on inflammatory pathways, and its broader potential to enhance patient outcomes and quality of life. This review brings together current findings on the mechanisms and clinical efficacy of acoustic therapy and proposes its integration into comprehensive treatment strategies for AR and CRS. We explore the role of the microbiome in the development of AR and CRS, the effects of acoustic therapy on inflammation, and its potential to improve patients' quality of life. This review advocates for acoustic therapy as a complementary or alternative treatment within respiratory medicine and highlights research gaps to establish its place in clinical practice.

## 2 Methods

A literature search was conducted using the following electronic databases: PubMed, Scopus, ScienceDirect, and CINAHL. The search covered publications from 1994 to 2025 to ensure a broad inclusion of relevant studies.

The search strategy employed a combination of keywords and MeSH terms including: “acoustic therapy”, “vibrational therapy”, “sound wave therapy”, “allergic rhinitis”, “chronic rhinosinusitis”, “nasal inflammation”, “nitric oxide”, “mucociliary clearance”, “microbiome”, “immune modulation”, “biofilm disruption”, “ultrasound therapy”, “mechanostimulation”, and “non-pharmacological treatment”.

Boolean operators (AND, OR) were used to refine searches and combine terms. Titles and abstracts of identified articles were screened for relevance, followed by full-text reviews to select studies addressing the mechanisms, clinical efficacy, immunological effects, and microbiome interactions of acoustic therapy in AR and CRS.

## 3 Pathophysiology of AR and CRS

The pathophysiology of AR and CRS is rooted in distinct but overlapping patterns of immune dysregulation. In AR, the immune response is heavily skewed towards a type 2 (T_h_2) bias, characterized by elevated levels of interleukin (IL)-4, IL-5, and IL-13 (17). This cascade of events drives the production of allergen-specific immunoglobulin E (IgE), leading to mast cell degranulation, eosinophil recruitment, and the hallmark symptoms of sneezing, itching, and nasal congestion. By contrast, CRS is characterized by a more complex interplay between type 1 (T_h_1), type 17 (T_h_17), and regulatory T-cell (T_reg_) pathways (18). In some patients, a skew towards type 1 and type 17 cytokines perpetuates neutrophilic inflammation, while in others, an impaired regulatory T-cell response fails to suppress persistent mucosal immune activation (19, 20). These imbalances ultimately compromise epithelial barrier integrity and sustain the chronic inflammation that defines CRS.

Recent high-throughput sequencing studies have provided new insights into the altered microbial ecosystems accompanying these immune derangements. Analyses of the bacterial communities lining the nasal passages consistently reveal a reduction in overall microbial diversity among patients with AR or CRS (21, 22). Beneficial commensals such as *Corynebacterium* and *Dolosigranulum* are significantly depleted, while potentially harmful pathogens, including *Staphylococcus aureus*, become more prevalent (23, 24). These shifts in microbial composition correlate with disease severity, suggesting that the loss of protective microbes may exacerbate mucosal inflammation and reduce the resilience of the nasal ecosystem to invading pathogens.

The consequences of nasal obstruction extend beyond the physical discomfort of congestion and blocked breathing, reaching into many aspects of daily life and mental health. Patients with persistent nasal blockage often report poor sleep quality, as evidenced by elevated scores on the Pittsburgh Sleep Quality Index (PSQI) (25). Difficulty breathing through the nose is also strongly correlated with heightened levels of anxiety and depression, as measured by the Zung Self-rating Anxiety/Depression Scale (SDS/SAS) (26), reflecting the significant emotional toll of chronic symptoms. Furthermore, health-related quality of life, assessed through instruments like the Sino-Nasal Outcome Test-22 (SNOT-22) and the EuroQol-5D (EQ-5D), is substantially diminished in these populations (27, 28), highlighting the widespread impact of AR and CRS on overall well-being.

Restoring microbial balance and modulating excessive inflammation could therefore offer a dual benefit: alleviating the physical symptoms of disease while also improving sleep, mood, and overall quality of life. Addressing both the immune and microbial factors underlying these conditions can lead to more comprehensive relief from nasal obstruction and the broader effects on mental health and well-being.

## 4 History and mechanisms behind acoustic therapy

Acoustic therapy, also known as vibrational sound therapy or acoustic resonance therapy (ART), is an ancient and evolving healing practice that uses sound and vibrations to restore balance and harmony within the body. Rooted in time-honored traditions, it has been used for centuries to promote relaxation and well-being, based on the principle that everything in the universe, including the human body, is in a constant state of vibration (29, 30). In modern times, acoustic therapy has also found a firm foundation in clinical and biomedical contexts. Often referred to as vibrational or mechanostimulation therapy in these settings, it has been applied across several medical fields, particularly in physiotherapy and pulmonology. Over the past few decades, vibrational therapy has been effectively employed to promote healing and improve physiological outcomes in various conditions.

Vibroacoustic Therapy (VAT) is a therapeutic approach that utilizes low-frequency sound vibrations to promote physical and emotional well-being. The development of VAT can be traced back to the 1960s, with significant contributions from Norwegian researcher Olav Skille. In 1968, Skille and British music therapist Juliette Alvin began exploring the potential of integrating low-frequency sound vibrations into traditional music therapy (31). Their collaboration laid the groundwork for what would later be known as VAT. VAT has been explored for its therapeutic effects in various conditions, including musculoskeletal and neurological disorders. Prior to VAT, therapeutic ultrasound, a form of acoustic therapy, has been used in physiotherapy since the mid-20th century to promote tissue healing, reduce pain, and improve circulation (32). This technique utilizes high-frequency sound waves to penetrate tissues, facilitating cellular repair and reducing inflammation. Ultrasound waves have been used for over six decades to treat musculoskeletal injuries, stimulate circulation, and enhance tissue regeneration (33).

In pulmonology, vibration therapy has proven beneficial in treating respiratory conditions. Whole-body vibration therapy (WBV) has been studied for its potential benefits in patients with chronic obstructive pulmonary disease (COPD). Research indicates that WBV can enhance exercise capacity and muscle function, improving the quality of life for individuals with COPD (34). Furthermore, chest physiotherapy techniques such as high-frequency chest wall oscillation (HFCWO) have improved mucus clearance in patients with cystic fibrosis and COPD (35, 36). These therapies work by delivering mechanical vibrations to the chest to mobilize mucus, improve airflow, and reduce symptoms related to airway obstruction. These prior clinical applications of acoustic and vibrational therapy in physiotherapy and pulmonology provide an essential foundation for understanding its potential in nasal and sinus health.

### 4.1 Mechanostimulation and nitric oxide production

**Figure 1:**
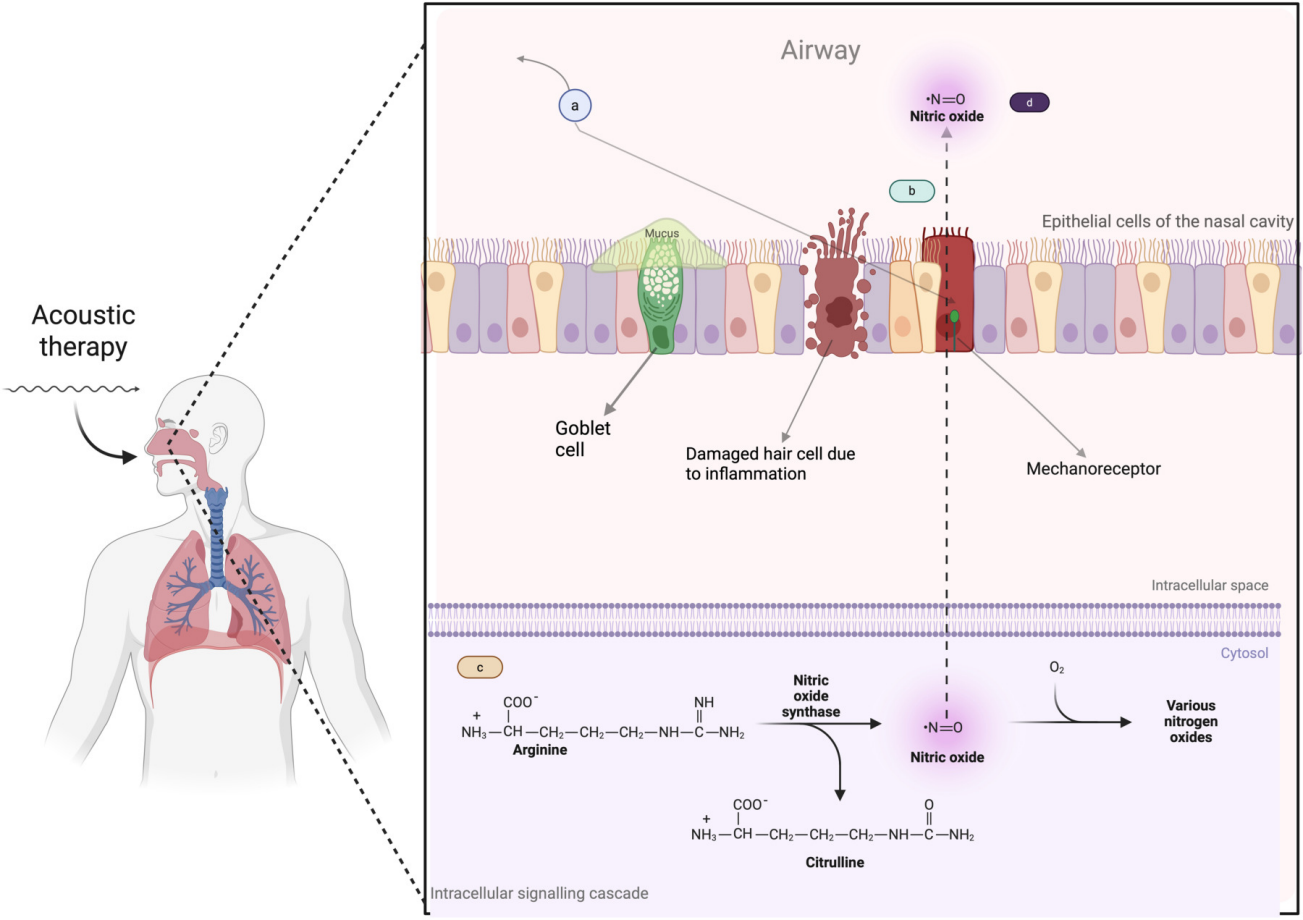
How acoustic therapy induces mechanostimulation and the production of nitric oxide. **(a)** Acoustic therapy generates sound waves that target nasal epithelial cells, initiating mechanostimulation. **(b)** The sound waves induce mechanical forces on the epithelial cells, causing deformation/stretching of the cell membrane, which activates mechanosensitive ion channels (mechanoreceptors). **(c)** Once the mechanosensitive channels are activated, intracellular signaling pathways are triggered, activating NO production pathways. **(d)** Mechanostimulation ultimately leads to an increased availability of NO. Created in BioRender. Alao, J. (2025) https://BioRender.com/e14n637.

Acoustic therapy uses vibrational energy applied to the nasal epithelium, which activates mechanosensitive ion channels in epithelial cells. This stimulation causes subtle deformation of the cell membrane and triggers intracellular signalling pathways (37, 38). One essential signaling cascade that mechanostimulation activates is the NO production pathway (Figure 1). This process begins with the amino acid L-arginine, converted into NO and L-citrulline by the enzyme nitric oxide synthase (NOS) (39). Several cofactors, including Nicotinamide adenine dinucleotide phosphate (NADPH), tetrahydrobiopterin (BH_4_), and calcium ions, are required for this reaction. Once produced, NO is a critical signaling molecule that regulates vasodilation, immune responses, and tissue regeneration. L-citrulline can be recycled back into L-arginine, allowing for sustained NO availability. This NO availability is essential to nasal and sinus health by promoting blood flow, enhancing mucosal defense, and reducing inflammation (40, 41).

The resulting increase in nasal NO is particularly important for enhancing mucociliary clearance. It accelerates the frequency of ciliary beat and coordinates the motion of the cilia, which improves the removal of mucus and trapped particles from the nasal passages and sinuses (42–46). Additionally, nitric oxide-induced vasodilation improves blood flow to the mucosa, reducing congestion and improving tissue oxygenation. Direct evidence for these effects comes from previous studies (12, 13), where humming significantly increased nasal nitric oxide concentrations. Similarly, ultrasonic-based therapies have demonstrated an augmentation in NO release in sinonasal tissues, further supporting this mechanism (47).

### 4.2 Biofilm disruption

In CRS, bacterial biofilms often form on the nasal and sinus epithelium, contributing to persistent infections. Acoustic therapy, through oscillatory pressure and targeted vibrations, can mechanically fracture the extracellular matrix of these biofilms, making the resident bacteria more susceptible to both nitric oxide and immune clearance (48).

*in vitro* studies have shown that ultrasound waves can effectively disrupt the biofilm integrity of common sinus pathogens, allowing antibiotics to penetrate more effectively (49, 50). Clinical pilot studies using pulsed ultrasound in patients with chronic rhinosinusitis have similarly reported reductions in biofilm density and improvements in bacterial clearance (15), highlighting the potential of acoustic therapy to aid in managing biofilm-associated infections.

### 4.3 Microenvironmental changes

Acoustic therapy can also induce transient changes in the physicochemical environment of the nasal cavity, creating conditions that promote the growth of beneficial commensal bacteria while inhibiting the overgrowth of pathogenic species. The vibrational energy gently agitates the mucus layer, subtly modifying local pH, humidity, and airflow dynamics (51). These changes influence the microbial community structure, with improved airflow and reduced mucus viscosity further enhancing oxygenation and temperature regulation within the sinuses. These microenvironmental shifts support the re-establishment of a balanced nasal microbiome, which may help prevent the overgrowth of harmful pathogens. As a result, acoustic therapy contributes to long-term sinonasal health by creating an environment that favors commensal bacteria and discourages the proliferation of opportunistic pathogens. This restoration of microbial balance is a promising avenue for future research in treating CRS and other nasal conditions.

## 5 Acoustic therapy and the nasal microbiome

### 5.1 Overview of healthy versus diseased microbiome

In a healthy nasal ecosystem, the microbiome is predominantly composed of a diverse range of commensal bacteria, such as *Corynebacterium*, *Dolosigranulum*, and *Streptococcus* (52), which play vital roles in immune regulation, mucosal protection, and overall respiratory health. These microorganisms coexist harmoniously, outcompeting potential pathogens for space and nutrients, while also producing metabolites that contribute to the integrity of the epithelial barrier. Additionally, fungi such as *Malassezia* and low levels of *Candida* species are part of the natural microbiome, residing without triggering inflammation (53, 54). However, in patients with AR or CRS, this balance is disrupted. There is a significant reduction in microbial diversity, with commensals like *Corynebacterium* being notably depleted. This depletion allows pathogens, such as *S. aureus* and *Pseudomonas aeruginosa*, to proliferate, exacerbating the disease (55–57). The fungal component also undergoes changes, with overgrowth of opportunistic fungi like *Candida* and *Aspergillus* linked to increased inflammation and symptom severity (58, 59). This shift in both bacterial and fungal populations, termed “microbiome dysbiosis” is implicated in the development and persistence of AR and CRS, highlighting the importance of a balanced nasal microbiome for maintaining sinus health.

### 5.2 Hypotheses for modulation by acoustic therapy

Acoustic therapy may restore microbial balance in the nasal cavity by modulating NO levels. NO has shown selective antimicrobial properties, capable of inhibiting the growth of pathogenic microorganisms while promoting the repopulation of beneficial commensals (60). Enhancing NO production through vibrational stimulation allows acoustic therapy to selectively target harmful pathogens while supporting the growth of protective microbial species, thereby promoting a healthier nasal microbiome. Another hypothesis suggests that acoustic therapy could disrupt bacterial biofilms often found in chronic sinus infections. Biofilms, formed by bacteria such as *P. aeruginosa* and *S. aureus*, act as a protective shield against immune responses and antibiotic treatments, enabling the bacteria to persist in the sinuses (61). Mechanical vibrations from acoustic therapy could potentially break down the biofilm matrix, making the bacteria more susceptible to immune clearance and antimicrobial agents. This disruption may occur without fully eliminating the beneficial microbes that form part of the healthy microbiome, ensuring that the microbial ecosystem is not entirely wiped out. Finally, acoustic therapy may influence the local microenvironment within the nasal cavity, altering factors such as pH, humidity, and airflow dynamics. These changes could create conditions that favor the growth of commensal bacteria while inhibiting the overgrowth of harmful pathogens, helping restore and maintain a balanced microbiome.

### 5.3 Proposed research approaches

Well-structured clinical trials should be conducted to explore acoustic therapy's effects on the nasal microbiome. Patients with moderate to severe AR or CRS would be randomized to receive either standard care or standard care combined with daily acoustic therapy for six weeks. Nasal swabs and brushings would be collected at baseline, immediately after the final treatment session, and at a three-month follow-up to capture both immediate and long-term microbiome shifts. High-throughput 16S rRNA sequencing could be used to characterize bacterial communities, while internal transcribed spacer (ITS) sequencing would map fungal populations at each time point (62). Key diversity metrics, such as alpha diversity (e.g., Shannon index) and beta diversity, could be analyzed to detect any shifts in microbial community structure. For example, the relative abundance of specific bacterial and fungal taxa such as *Corynebacterium*, *Dolosigranulum*, *S. aureus*, and *Candida* could be tracked over time. These microbial changes can then be correlated with clinical outcomes, including symptom scores and objective measures of nasal airflow. This approach would provide information on how acoustic therapy influences both the bacterial and fungal components of the nasal microbiome and whether these changes contribute to improvements in clinical outcomes. Investigating the relationship between microbial shifts and symptom relief could shed light on the potential of acoustic therapy to restore a balanced nasal ecosystem and support overall sinonasal health. Additionally, the research could provide further understanding of how modulation of the nasal microbiome impacts immune function, inflammation, and symptom relief in AR and CRS patients.

## 6 Immunological impact of acoustic therapy

### 6.1 Modulation of inflammatory cytokines

Acoustic therapy has shown potential in modulating inflammatory responses, particularly through mechanisms such as photobiomodulation (PBM) and low-intensity pulsed ultrasound (LIPUS). Studies have demonstrated that PBM and LIPUS can downregulate pro-inflammatory cytokines, such as IL-1β and IL-6, while simultaneously upregulating anti-inflammatory cytokines like IL-10 (63, 64). This shift suggests that acoustic therapy can alter the immune response, favouring a more balanced, anti-inflammatory state. In conditions like AR and CRS, where chronic inflammation is a hallmark, such modulation could provide significant therapeutic benefits.

In AR and CRS, elevated levels of pro-inflammatory cytokines like IL-1β, IL-6, and TNF-α are commonly observed (65, 66). These cytokines are key in driving the inflammation, tissue damage, and remodelling seen in these conditions. The ability of acoustic therapy to modulate these cytokines suggests it could be instrumental in alleviating symptoms and improving patient outcomes. Reducing the levels of these inflammatory mediators through acoustic therapy could help control the chronic inflammation underlying symptoms of both AR and CRS, offering a valuable addition to current treatment approaches.

### 6.2 Impact on immune cell populations

Beyond cytokine modulation, acoustic therapy may influence the balance of immune cell populations in the nasal mucosa. Research has shown that therapies that enhance IL-10 production are linked to reduced T_h_2 cell activity and increased regulatory T cells (T_regs_) (67). This shift is important, as T_h_2 cells are typically associated with allergic responses, while T_regs_ help maintain immune tolerance to allergens (67). Promoting this balance through acoustic therapy may reduce the allergic response and enhance tolerance, an especially relevant benefit for AR patients who experience hypersensitivity to environmental allergens.

Moreover, acoustic therapy's influence on immune cells could also enhance mucosal immunity. Promoting a more balanced immune environment may improve the nasal mucosa's ability to respond effectively to pathogens and allergens. This could reduce the frequency and severity of AR and CRS exacerbations, potentially preventing recurrent infections and the chronic inflammation that exacerbates symptoms.

### 6.3 Enhancement of mucosal barrier function

Acoustic therapy's effects on immune cell modulation and cytokine levels may also contribute to strengthening the nasal mucosal barrier. Chronic inflammation and tissue damage in AR and CRS can weaken the mucosal lining (68, 69), making it more susceptible to pathogen entry. Reducing inflammation and promoting the repair of epithelial cells through acoustic therapy may help restore the integrity of the mucosal barrier. A robust mucosal barrier is crucial for preventing the invasion of pathogens and maintaining overall nasal health. This repair and strengthening of the mucosal lining could lead to improved respiratory function and a reduced risk of secondary infections, which are common in patients with CRS and AR.

### 6.4 Proposed research approaches

To further understand the immunological effects of acoustic therapy, well-designed clinical trials are essential. These studies could focus on measuring changes in cytokine levels, immune cell populations, and mucosal barrier function before and after acoustic therapy. The ability to track these immunological markers will provide information into how acoustic therapy influences the immune system at a cellular level. Moreover, correlating these changes with clinical outcomes, such as symptom severity and overall quality of life, will help assess the true therapeutic potential of acoustic therapy in treating AR and CRS.

Longitudinal studies could be beneficial in evaluating the sustained effects of acoustic therapy over time. Mechanistic investigations could also explore the cellular and molecular pathways through which acoustic therapy modulates immune responses, and how these changes might correlate with clinical improvements. Randomized controlled trials (RCTs) would be instrumental in validating these findings and establishing acoustic therapy as a standard, non-pharmacological adjunct to current treatment regimens for CRS and AR.

Biomarker monitoring is a crucial component of these studies. Specifically, measuring cytokines such as IL-4, IL-5, IL-13, IL-10, and IFN-γ, along with chemokines like CXCL8 (IL-8), can provide insights into the inflammatory processes at play. These biomarkers are often used to characterize the type of inflammation, such as T_h_2-skewed or regulatory immune profiles and to assess how these responses evolve over the course of treatment. Samples could be collected either from nasal lavage fluid or blood to obtain these measurements. Nasal lavage involves rinsing the nasal cavity with saline to collect mucosal secretions, which reflect local airway inflammation. Blood samples, processed to obtain either serum or plasma, provide a systemic view of circulating biomarker levels. In longitudinal studies, samples are usually taken before and after therapy to track biomarker fluctuations in response to intervention.

Quantifying these biomarkers requires sensitive and reliable immunoassay techniques. The enzyme-linked immunosorbent assay (ELISA) is an established method that uses antibody-based detection to quantify specific proteins (70). It is highly sensitive, capable of detecting cytokine concentrations as low as a few picograms per milliliter. For example, IL-8 levels can be measured in nasal or serum samples using ELISA with a detection threshold around 7.5 pg/ml and a quantifiable range extending to approximately 2,000 pg/ml (71). However, standard ELISAs are limited to measuring one analyte per assay, which can be time-consuming when profiling multiple markers.

To address this limitation, many studies now employ multiplex cytokine assays. These advanced platforms such as bead-based flow cytometric arrays or electrochemiluminescence-based systems allow simultaneous detection of numerous cytokines within a single sample (72–74). They maintain the high sensitivity of single-analyte methods while significantly increasing throughput. This multiplexing capability is especially valuable in inflammatory research, as it enables a more integrated understanding of the complex and dynamic cytokine networks involved in disease progression and therapeutic response.

## 7 Broader impacts on well-being

**Figure 2:**
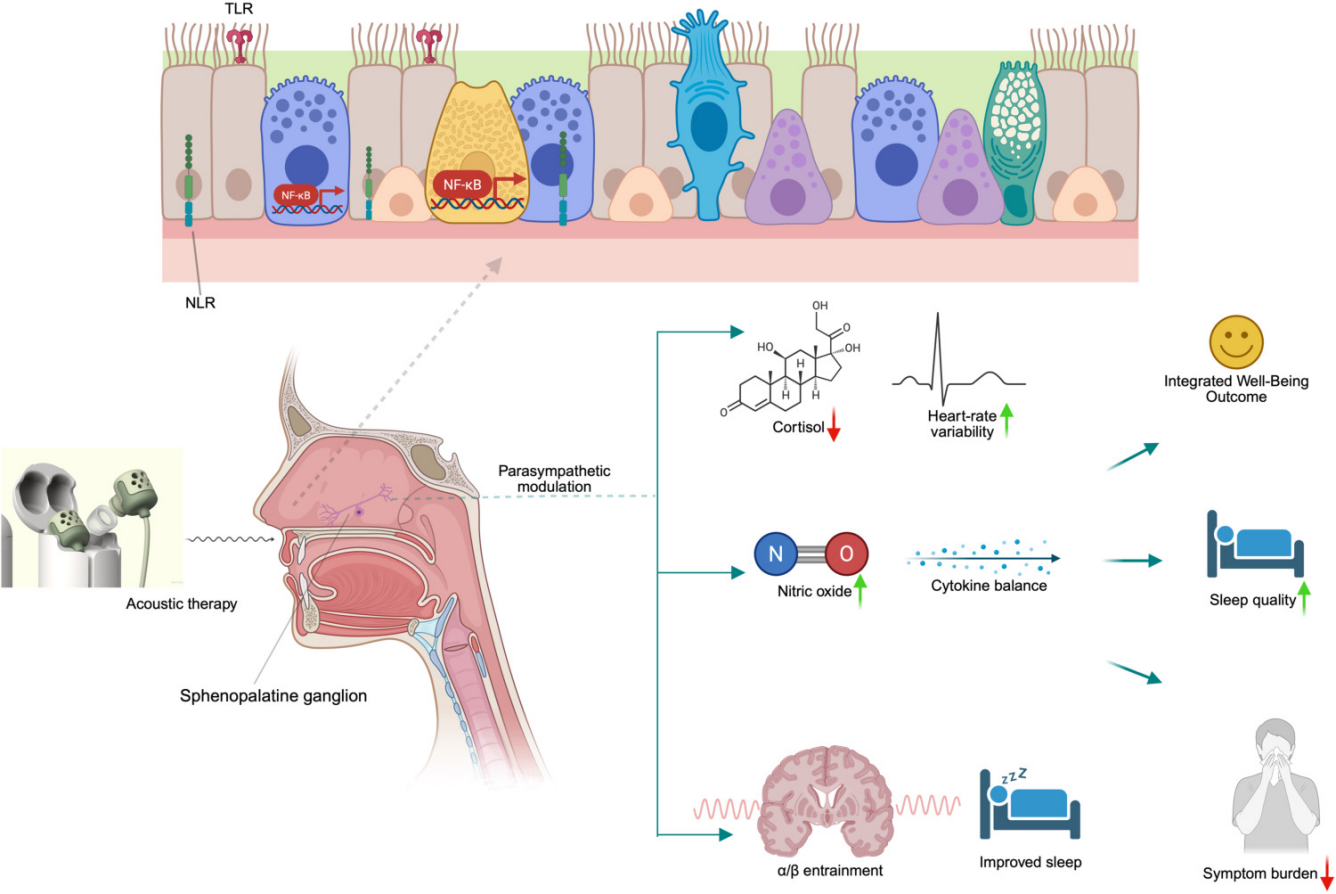
Mechanistic model of acoustic therapy impacts on nasal-autonomic-mental physiology. Acoustic waves stimulate the sphenopalatine ganglion within the nasal mucosa, triggering parasympathetic up-regulation and local nitric oxide release. This dual autonomic–immune modulation supports improved sleep, reduced inflammatory cytokines, and enhanced mood through neuro-entrainment pathways, culminating in integrated well-being outcomes. Created in BioRender. Alao, J. (2025) https://BioRender.com/bk8urjj.

Acoustic therapy has the potential to significantly enhance overall well-being, extending beyond its primary focus on improving respiratory health (Figure 2). Well-being encompasses physical health and psychological and neurological aspects, all of which are vital to quality of life (75). Studies suggest that acoustic therapy may improve respiratory function while also offering psychological benefits such as enhanced mental clarity and emotional stability (76, 77). These improvements can create a positive feedback loop, where physical health enhancements support psychological well-being and vice versa. In addition to alleviating symptoms in conditions like AR and CRS, which are often associated with chronic discomfort and psychological strain, acoustic therapy may contribute to a more balanced emotional state. This holistic impact on well-being suggests that acoustic therapy could be a valuable adjunct to traditional treatments, supporting both the physical and emotional aspects of recovery.

A proposed mechanism for these benefits involves acoustic therapy's potential to modulate the autonomic nervous system, specifically through parasympathetic pathways. Although studies have primarily focused on other areas, evidence suggests that vibrational therapy can significantly impact autonomic regulation. Acoustic therapy has been shown to modulate parasympathetic innervation via the sphenopalatine ganglion (SPG), a key structure involved in autonomic control. This ganglion is essential in regulating parasympathetic activity, and therapies targeting it have been used for conditions such as headaches and facial pain (78, 79). Research on autonomic dysfunction indicates that interventions which balance autonomic activity can reduce inflammation and improve mucosal function (80, 81), offering potential benefits for sinonasal conditions. Although further research within ENT and sinonasal health is required to understand this mechanism fully, preliminary evidence supports the idea that acoustic therapy could modulate autonomic activity and help alleviate AR and CRS symptoms.

### 7.1 Sleep quality

Acoustic therapy has been shown to improve sleep quality by enhancing nasal airflow and reducing congestion, thereby enabling more comfortable and uninterrupted breathing during sleep (82, 83). This benefit is significant for individuals with sleep apnoea or chronic nasal congestion, where improving airflow can significantly enhance sleep architecture. Studies suggest that the production of NO stimulated by acoustic therapy is linked to better regulation of circadian rhythms (84). NO plays a role in modulating the sleep-wake cycle and vascular tone in areas of the brain involved in sleep regulation (85).

A prospective study involving 25 participants with obstructive sleep apnea (OSA) found that the use of an ART headband system improved continuous positive airway pressure (CPAP) adherence and reduced nasal symptoms. Participants who used the ART headband experienced increased CPAP usage hours and reported better sleep quality (86). Furthermore, a pilot study involving 20 participants with chronic insomnia found that personalized ART using a headband system significantly improved sleep quality. After four weeks of treatment, nearly half of the participants achieved a clinically significant reduction in insomnia severity, and adherence to the therapy was 100% (87).

These findings suggest that acoustic therapy, particularly ART, may offer a non-pharmacological approach to improving sleep quality in individuals with nasal congestion, sleep apnea, and insomnia. To assess these effects more rigorously, polysomnography or validated questionnaires such as the Pittsburgh Sleep Quality Index (PSQI) (88) should be used to monitor sleep quality before and after treatment.

### 7.2 Stress and anxiety

Acoustic therapy promotes relaxation by influencing the autonomic nervous system, specifically through parasympathetic activation. This activation helps reduce stress levels and aids in recovery from sympathetic overdrive (89), which is common in conditions like AR and CRS. Research indicates that the vibration therapy associated with acoustic treatment can lower cortisol levels (90), a key stress hormone, and improve heart rate variability (HRV) (91), an indicator of parasympathetic function. Enhancing parasympathetic activity through vibration of the SPG via acoustic therapy could contribute to stress management.

A pilot trial involving university students exposed to LFS found significant increases in parasympathetic nervous system activity, as evidenced by HRV metrics such as Root Mean Square of Successive Differences (RMSSD) and high frequency components. This was associated with alleviating subjective stress responses and muscle tension, suggesting LFS as a potential tool for stress management in educational settings (91). Another study utilizing a vibroacoustic device to deliver low-frequency vibrations reported reductions in heart rate and improvements in HRV, indicating enhanced parasympathetic activity. Participants also experienced decreased pain and tension, along with increased relaxation and mental clarity, highlighting VAS's potential in stress and anxiety management (89). HRV monitoring and salivary cortisol assays in trials could provide information into the extent of acoustic therapy's effects on stress and autonomic function.

### 7.3 Mood and cognitive function

Acoustic therapy may improve mood and cognitive function by enhancing breathing and NO signaling, both of which benefit cerebral perfusion and brain function (92, 93). Improved breathing eases nasal congestion, allowing for better oxygen delivery to the brain, while NO has been shown to influence neurotransmission and enhance brain perfusion, which can improve mood regulation and cognitive clarity (94, 95). Furthermore, NO plays a vital role in neurotransmission and neural repair, potentially contributing to alleviating depressive symptoms (96, 97), although its exact mechanisms require further exploration.

Acoustic therapy also helps modulate brainwave activity and promote neural plasticity through vibrational or auditory stimulation. This stimulation aids in stabilizing mood by entraining brainwave frequencies associated with relaxation and mental clarity (98). For example, rhythmic acoustic stimulation has been shown to enhance slow oscillation activity during sleep (99), which is linked to memory consolidation and mood regulation. Furthermore, auditory stimulation can facilitate neural plasticity by engaging the cholinergic and noradrenergic systems, which are involved in learning, memory, and mood regulation. Vagus nerve stimulation paired with auditory stimuli can induce long-lasting changes in auditory cortical responses, leading to improved auditory perception and potentially alleviating symptoms of depression (100).

A study utilizing 10 Hz acoustic neurostimulation reported significant reductions in symptoms of stress, depression, and anxiety, along with improvements in sleep quality, as assessed by the DASS-21 and PSQI scales (101). These effects are particularly beneficial for individuals experiencing mental fog or depressive symptoms, common in conditions like AR and CRS, where nasal congestion and inflammation often exacerbate cognitive dysfunction and mood instability. Additionally, the activation of olfactory nerves during acoustic therapy can indirectly influence the limbic system, which governs emotions and mood, further contributing to stress and mood regulation.

Given the limited direct evidence on acoustic therapy for depression related to AR and CRS, targeted research in these specific conditions remain a priority. To rigorously assess the impact of acoustic therapy on mood and cognitive function, tools such as the Hospital Anxiety and Depression Scale (HADS) or the Beck Depression Inventory (BDI) (102), alongside cognitive tests, can be used pre- and post-intervention to evaluate improvements in mood and cognitive clarity.

### 7.4 Patient-reported outcome measures

Nasal congestion in AR and CRS often results from inflammatory processes that lead to mucosal swelling and increased vascular permeability, contributing to the sensation of nasal obstruction. While facial pain, pressure, and headaches are common symptoms in CRS, they are not driven by direct mechanical blockage of the sinuses but by neurogenic inflammation and activation of trigeminal nociceptors in response to inflammatory mediators, edema, and altered mucosal sensory signaling (103). These symptoms may not always correlate with the extent of sinus pathology, suggesting that factors beyond sinus blockage contribute to these sensations. This discomfort is often aggravated by bending forward or lying down, as elevated sinus pressure stretches the sinus walls and surrounding tissues, leading to nociceptive pain. Chronic inflammatory swelling of the mucosa activates pain receptors through neurogenic inflammation pathways (104), which can exacerbate the sensation of facial pain. Acoustic therapy has demonstrated significant improvements in PROMs, including reductions in nasal congestion, pain, and pressure and increased patient satisfaction and safety. These benefits have been observed across various time frames, from immediate post-treatment effects to longer-term outcomes.

A pilot study using multimodal vibration techniques, such as ultrasound and acoustic resonance, have reported significant reductions in CRS-related facial pain, with improvements in SNOT-22 scores exceeding the clinically meaningful threshold by around 14 points (105). Furthermore, the modulation of autonomic pathways, particularly through the SPG, holds promise for neurogenic modulation of pain perception (106). Another prospective single-arm observational study investigated the efficacy of a device combining acoustic vibration with oscillating expiratory pressure in patients reporting “sinus headache” without evidence of CRS. Over a 4-week period, patients used the device twice daily. The study found significant improvements in pain metrics: the Visual Analog Scale (VAS) for facial pain decreased from 59.6 ± 15.7 to 34.6 ± 21.7 (*p* < .001), the Brief Pain Inventory-Short Form (BPI-SF) mean pain score improved from 4.4 ± 2.0 to 2.9 ± 1.9 (*p* = .007), and the McGill Pain Questionnaire-Short Form (MPQ-SF) total score decreased from 12.2 ± 6.5 to 6.5 ± 5.2 (*p* < .001) (107).

Specific PROMs can be utilized to understand acoustic therapy's impact better. Tools like the SNOT-22 for sinonasal symptoms (108), the EQ-5D for quality of life (27), the PSQI for sleep quality (88), and the HADS for anxiety and depression will provide important data on the effects of acoustic therapy. Correlating changes in these measures with clinical outcomes allows researchers to gain a more comprehensive understanding of acoustic therapy's overall impact on patient well-being.

## 8 Clinical evidence: synthesis and critique

### 8.1 Symptom relief trials

Several clinical trials have explored the efficacy of acoustic therapy in alleviating symptoms of AR and CRS (Table 1). One notable study used a device employing acoustic vibration and oscillating expiratory pressure on 14 participants, reporting significant improvements in nasal congestion (*p* < 0.05) and ease of breathing (*p* < 0.05) (82). Despite these promising results, the small sample size limits the generalizability of the findings. A larger trial involving 40 participants that combined acoustic vibration with oscillating expiratory pressure showed a 31% increase in peak nasal inspiratory flow (PNIF) and reductions in Total Nasal Symptom Score (TNSS) and SNOT-22 scores (109). However, this study's lack of a control group and moderate sample size weakens the conclusions, highlighting the need for larger, more rigorous trials with randomization and control groups. Another study, involving 52 adults, found that acoustic therapy reduced nasal congestion sub-scores and composite TNSS scores over a two-week period, with an 80.8% response rate in the treatment group (110). Although the use of a sham control enhances the study's validity, the short duration limits any conclusions about long-term efficacy. Notably, none of these studies directly addressed microbiome or immunological endpoints, which remain a gap in the existing literature.

**Table 1:** A comparison of studies on vibration-based interventions for nasal congestion .

Population	Intervention	Comparison	Outcome	Time	Statistical significance	Study limitations	Reference
Adults with chronic nasal congestion	Nasal airflow oscillation device	No comparison/placebo	Reduction in nasal congestion and improved airflow	Cross-sectional study	Yes (PNIF ↑ from 84.8 to 99.0 L/min; VAS congestion ↓, *p* < 0.05)	Small sample (*n* = 21); no control group; very short duration (20 min single session); not blinded; preliminary findings require further study	(111)
Adults with nasal congestion	SinuSonic device (acoustic vibration + oscillating expiratory pressure)	Sham or no device	Improved nasal congestion and airflow; device found safe and tolerable	Cross-sectional study	Yes (VAS congestion *p* = 0.002; ease-of-breathing *p* = 0.003)	Small pilot sample (*n* = 14); no placebo control and no blinding (possible placebo effect); single application only (acute effect); conflict of interest (device inventor involved)	(82)
Adults with acute nasal congestion	Resonant vibration therapy of the sinonasal cavities (personalized frequency)	No control group	Effectiveness of resonant vibration therapy in reducing nasal congestion	Cross-sectional study	Yes (TNSS score improved from 4.95 to 2.57 after 20 min, *p* < 0.0001)	Small sample (*n* = 21); uncontrolled pre-post pilot; very short-term (two 10 min treatments in one visit); not blinded; outcomes mostly subjective (no objective airflow measure)	(115)
Adults with rhinitis (nasal congestion)	Acoustic resonance therapy (sound & vibration via headband)	Sham-controlled (placebo device)	Reduced nasal congestion and overall rhinitis symptoms; treatment deemed safe	2 weeks (twice daily)	Yes (greater congestion reduction vs. sham, *p* = 0.008; TNSS improvement *p* = 0.027)	Short trial duration (2 weeks); moderate sample size (*n* = 52); some investigators affiliated with device manufacturer (potential bias); long-term efficacy and durability of effect not assessed (no data beyond 2 weeks).	(110)
Adults with chronic nasal congestion	Combined acoustic vibration + oscillating expiratory pressure device	No control or sham	Significant reduction in nasal congestion; improved sinus ventilation and airflow	5 weeks (with 2- and 5-week follow-up)	Yes (PNIF ↑31% at 2 weeks, *p* < 0.001; nasal congestion VAS from 5.8 to 2.6 at 5 weeks, *p* < 0.001)	Small sample (*n* = 40); no control/sham (within-subject baseline comparison only); not blinded; short follow-up (only 5 weeks); results preliminary; further studies needed to confirm efficacy	(109)

PNIF, peak nasal inspiratory flow; VAS, visual analog scale; TNSS, total nasal symptom score.

In addition to these early studies, recent trials in diverse populations have bolstered clinical evidence for acoustic or vibratory therapy in clearing nasal congestion. For example, a New Zealand study evaluated a nasal airflow oscillation device in 21 adults with chronic nasal congestion and reported a significant acute improvement in nasal airflow. After a single 20 min session, average PNIF increased from ∼85 L/min to ∼99 L/min (≈17% improvement, *p* < 0.05) with a corresponding decrease in congestion severity on a visual analogue scale (111). Patients also reported immediate relief of sinonasal pressure and drainage; no change in olfaction was noted over this short term. This pilot was uncontrolled and focused on immediate post-treatment effects; however, it demonstrated that even brief acoustic interventions can produce measurable decongestion.

Another line of evidence comes from a Finnish study targeting non-allergic rhinitis that examined a kinetic oscillation stimulation (KOS) device that mechanically vibrates the nasal cavity via an inflatable intranasal balloon. In a cohort of 49 patients with chronic idiopathic (vasomotor) rhinitis, a single KOS treatment (10 min per nostril) led to significant and durable symptom relief (112). At 12 months post-treatment, objective nasal airflow improved substantially (PNIF increased from ∼80 to 100 L/min, *p* < 0.005) and patient-reported congestion scores (NOSE and TNSS) were significantly reduced compared to baseline. Notably, this improvement persisted at one year without additional treatments, suggesting a prolonged benefit in nasal patency for non-allergic rhinitis patients. Although this was an open observational trial, its longer follow-up underscores the potential for sustained congestion relief from a single oscillatory intervention.

A randomized controlled trial of *Bhramari pranayama* (a yoga breathing exercise that produces humming vibrations) in 60 patients with CRS showed significant symptomatic improvement, including reduced congestion-related scores, compared to controls (113). This supports the concept that acoustic vibration of the sinonasal cavity, even through simple humming, can yield clinical benefits in chronic sinus conditions. Likewise, a Swedish randomized trial of the KOS therapy in 29 patients with non-allergic rhinitis demonstrated a reduction in patient-reported nasal stuffiness at 2 weeks post-treatment (114). Interestingly, in that study the subjective improvement occurred despite no significant change in PNIF (objective airflow), and only the self-administered treatment group (as opposed to physician-administered) achieved statistically significant relief. This inconsistency between perceived congestion relief and objective airflow highlights the complex nature of nasal obstruction symptoms and the potential influence of placebo effect or neural modulation.

These positive findings must be interpreted with caution. Many trials to date have been small-scale, short-term, and in some cases lacked rigorous controls or blinding, which diminishes the strength of their conclusions. The preponderance of published positive outcomes also raises the possibility of publication bias, whereby studies with neutral or negative results may be underreported. To date, no peer-reviewed trial has prominently reported a lack of symptom improvement with acoustic therapy, though some secondary outcomes have shown no significant change, for example, one pilot study noted no improvement in olfactory function after short-term acoustic treatment, and an RCT reported no objective PNIF gain despite subjective relief (114). Even the investigators of initial studies have cautioned that while results appear promising, the small sample sizes and brief follow-up make it challenging to generalize the findings. The newer trials echo these limitations. The aforementioned 21-patient study, for instance, had no control group and only evaluated immediate effects (111), and the 49-patient Finnish KOS study was unblinded and lacked a sham intervention (112).

Furthermore, heterogeneity in study design and patient populations (e.g., allergic rhinitis vs. non-allergic rhinitis, sinusitis vs. simple congestion) makes it challenging to compare outcomes directly. The mechanisms of acoustic vs. mechanical oscillation also differ slightly between devices (frequency, mode of delivery), which could influence efficacy. Therefore, the current evidence base should be considered preliminary. Larger, multi-center RCTs with appropriate sham controls are needed to validate these results and ensure that the observed benefits are not due to placebo effect or bias. Such trials could also examine longer-term efficacy and include objective endpoints (e.g., mucosal inflammation markers or microbiome changes) to determine whether acoustic therapy provides sustained, disease-modifying benefits beyond short-term symptom relief.

### 8.2 Safety and tolerability

In terms of safety, most studies report that acoustic therapy is generally well-tolerated. A study involving a vibrational headband device in 50 patients showed significant improvements in nasal symptoms, with reductions in TNSS and nasal congestion sub-scores after two 10-minute cycles (115). Participants also reported reduced facial pain, evidenced by lower visual analogue scale (VAS) scores (*p* < 0.05). This result suggests that acoustic therapy may be a safe and effective non-pharmacological treatment for nasal congestion and associated symptoms. However, most of the studies reviewed have not included long-term follow-up, indicating the need for more extended research to evaluate the durability of benefits and to monitor any potential long-term adverse events that may arise from sustained use.

Reassuringly, no serious device-related adverse effects have been reported in the published trials so far. For example, researchers noted no intervention-related severe or moderate adverse events in the sham-controlled 52-patient trial of acoustic resonance therapy (110). Across the various studies, side effects have generally been minor. In the short-term oscillation device study, no patients experienced any nosebleeds or mucosal injury after use (111). Similarly, the year-long KOS study reported no major complications among the 49 treated patients (112, 116), and biologics have demonstrated powerful anti-inflammatory effects, though at a much higher cost (8). The lack of direct comparison makes it difficult to determine whether acoustic therapy could serve as a viable alternative or if it might be more suitable as a complementary approach.

Many current studies are limited by short treatment durations, small sample sizes, and a lack of control groups that would provide more reliable data. To address these shortcomings, it is essential that we move toward large, well-designed RCTs that directly compare acoustic therapy with INCS and biologics. These studies could also explore deeper questions, such as how acoustic therapy might influence cytokine profiles, immune responses, and the composition of the nasal microbiome, alongside conducting thorough cost-effectiveness analyses to understand how it compares to current therapies.

**Figure 3:**
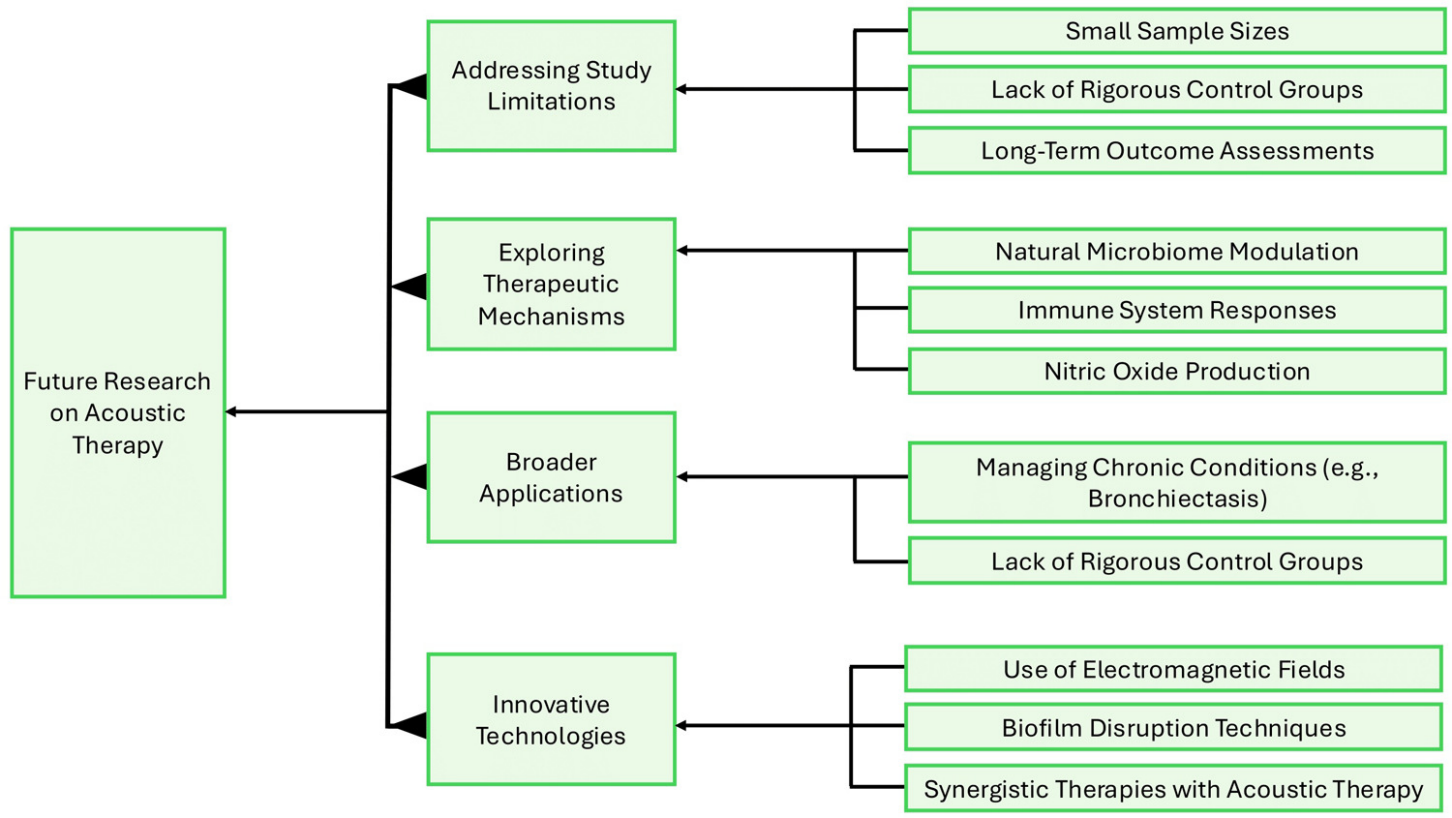
Conceptual framework for future research on acoustic therapy in AR and CRS.

To guide this next phase of research, a conceptual framework can help organize our approach (Figure 3). It could focus on addressing study limitations by ensuring larger sample sizes, including rigorous control groups, and tracking long-term outcomes. We must also explore the therapeutic mechanisms of acoustic therapy, particularly how it influences the nasal microbiome, immune responses, and NO production. Another important area to investigate is expanding the scope of trials to include other chronic airway conditions, like bronchiectasis, and ensuring the studies reflect the diversity of affected populations. Incorporating innovative technologies into research could be valuable, such as combining acoustic therapy with electromagnetic fields or adjunct pharmacotherapies to enhance overall effectiveness. Following this roadmap enables future research to bridge existing evidence gaps, clarify the biological effects of acoustic therapy, and define its role within the broader treatment landscape for AR and CRS.

## 9 Conclusion

Acoustic therapy has shown promising potential as a non-pharmacological treatment for nasal congestion, particularly in individuals with AR and CRS. Early clinical trials indicate significant symptom relief, including improved nasal airflow and reduced nasal congestion, with good short-term safety profiles. Despite these positive findings, the evidence base remains preliminary, with several studies limited by small sample sizes, short durations, and methodological concerns. Further rigorous, multi-center RCTs are needed to validate these findings, address long-term efficacy, and explore potential mechanisms underlying acoustic therapy benefits. Additionally, research could consider the integration of objective biomarkers and long-term follow-up to assess the durability of effects. As the field progresses, acoustic therapy could serve as an effective adjunct or alternative to traditional treatments, offering both physical and psychological benefits to patients suffering from nasal congestion.
